# Comparing the efficacy of different types of exercise therapy in patients with essential hypertension: a systematic review and network meta-analysis

**DOI:** 10.3389/fcvm.2025.1604112

**Published:** 2025-10-20

**Authors:** Longcheng Liu, Jiale Wang, Xiaodi Ji, Anqi Wang, Shuo Yang, Xiaojian Chi, Lingqian Hu, Baolin Zhang, Lihong Ma

**Affiliations:** ^1^ Fuwai Hospital, Chinese Academy of Medical Sciences and Peking Union Medical College, Beijing, China; ^2^ Second Affiliated Hospital of Xi'an Jiaotong University, Xi'an Jiaotong University, Xi'an, China

**Keywords:** aerobic exercise, Baduanjin, hypertension, Tai Chi, traditional Chinese exercise

## Abstract

**Objective:**

To compare the efficacy of different types of exercise modalities such as Tai Chi, Baduanjin, and Aerobic exercise in the treatment of hypertension.

**Methods:**

This article retrieved randomized controlled trials (RCTs) from the establishment of databases such as CNKI, WanFang, VIP, CBM, PubMed, Embase, Web of Science, and Cochrane Library to December 9, 2024. After assessing the risk of bias using the Cochrane risk of bias tool, a network meta-analysis was performed using Rev Man 5.4 and Stata 15 software. The primary outcome indicator of this article is Ambulatory Blood Pressure Measurement (ABPM), which includes 24-h average systolic blood pressure (SBP) and 24-h average diastolic blood pressure (DBP).

**Results:**

A total of 63 RCTs meeting the criteria were included, involving 5,663 patients. The network meta-analysis results showed that compared with the control group, Light-intensity continuous training (LIAE)+antihypertensive medication [WMD = −1.82, 95%CI(−2.93, −0.71)] was the most effective intervention for lowering SBP. For lowering DBP, Wuqinxi + antihypertensive medication [WMD = −2.78, 95% CI (−4.1,−1.45)] was the most effective intervention. And the differences were statistically significant (*P* < 0.05).

**Conclusion:**

Exercise therapy combined with antihypertensive drugs may achieve a more significant blood pressure-lowering effect, and LICT, Wuqinxi, and Liuzijue may be higher priority options.

**Systematic Review Registration:**

http://wwwcrdyork.ac.uk/PROSPERO/, PROSPERO CRD42024626332.

## 1 Introduction

Hypertension is one of the most significant health issues globally. It has a high prevalence rate and is highly predisposed to giving rise to complications such as cardiovascular diseases and chronic kidney diseases. It is also one of the major factors contributing to the reduction of the average lifespan (1, 2). Currently, the prevalence of hypertension continues to increase, and the overall prevalence rates of hypertension in males and females are relatively similar (1). In a global epidemiological survey, high systolic blood pressure (SBP) was identified as the primary risk factor for mortality (accounting for 10.4 million deaths) and disability-adjusted life years (DALYs) (amounting to 218 million) (3). Hypertension is a condition that can potentially be reversed. The traditional treatment mainly relies on pharmacotherapy, yet it is associated with multiple adverse effects and often fails to achieve satisfactory blood pressure control (4, 5). With the advancement of concepts in the diagnosis and treatment of hypertension, lifestyle interventions such as health education, dietary modification, weight management, and stress alleviation have gradually gained popularity (6). In recent years, lifestyle intervention using exercise therapy as an auxiliary method to control blood pressure has gradually gained attention and been recommended by multiple guidelines. On the basis of basic drug treatment, selecting an appropriate exercise therapy for intervention can reduce the side effects of drugs and achieve a better blood pressure control effect (7).

As a component of physical therapy, exercise therapy refers to a lifestyle that involves regular engagement in various forms of physical exercise—an approach that can also serve as a preventive measure against diseases. Notably, this therapy holds value across all stages of hypertension management, as physical exercise can be utilized for the prevention, treatment, and ongoing control of the condition (8). Beyond conventional exercise modalities like aerobic exercise, resistance training, and high-intensity interval training (HIIT), traditional Chinese exercises—including Tai Chi (TC), Baduanjin (BDJ), and Wuqinxi (WQX)—also form key components of exercise therapy. What makes this therapy particularly practical is its inherent advantages: it is easy to implement, low in cost, and associated with minimal adverse effects. These traits have allowed it to be widely applied in the prevention and treatment of a variety of diseases, with its most extensive use seen in the adjuvant treatment of cardiovascular diseases (6, 9).

Currently, numerous studies on the treatment of hypertension with exercise have demonstrated that exercise therapy is highly effective in reducing blood pressure in patients with hypertension (10, 11). However, there is a lack of studies comparing the therapeutic effects of different types of exercise, including aerobic exercises of varying intensities, resistance exercises, yoga, and Traditional China exercises. Moreover, in previous research, the comparison between traditional qigong exercises and conventional exercise therapy has often been overlooked. This article aims to compare the therapeutic effects of different types of exercise on patients with hypertension, with the expectation of providing a reference for the clinical management of hypertension.

## 2 Methods

The method used to conduct this systematic review has been previously published in a registered protocol [PROSPERO registration: CRD42024626332] (Available from: http://wwwcrdyork.ac.uk/PROSPERO/). The content of the review followed the Preferred Reporting Items for Systematic Reviews and Meta-Analyses (PRISMA) (Supplementary Material 2).

### 2.1 Databases and retrieval strategy

In this article, a systematic search was conducted in databases such as CNKI (China National Knowledge Infrastructure), Wanfang Data, VIP (Chongqing VIP Information), Sinomed, Embase, Web of Science, PubMed, and Cochrane Library. The search time range was from the establishment of these databases to December 9, 2024. The search strategy combined MeSH (Medical Subject Headings) terms and free terms, and was adjusted according to the search rules of different databases. The specific Chinese search terms included “exercise therapy”, “Baduanjin”, “Taijiquan”, “Wuqinxi”, “Yoga”, “High-Intensity Interval Training”, “High-Intensity Continuous Training”, “traditional qigong exercises”, “hypertension”, etc.; the English search terms included “Hypertension”, “TaiJi”, “Baduanjin”, “Wuqinxi”, “Qigong”, “kinesitherapy”, “High-Intensity Interval Training”, “resistance training”, etc. Taking CNKI and PubMed as examples, the search formulas are shown in Table 1.

**Table 1:** Search strategy for the PubMed database.

Query	Search term
#1	Hypertension [Mesh]
#2	((((((Hypertension[MeSH Terms]) OR (Hypertension[Title/Abstract])) OR (Hypertension[Title/Abstract])) OR (Hypertension[Title/Abstract])) OR (High Blood Pressure[Title/Abstract])) OR (High Blood Pressures[Title/Abstract]))
#3	#1 OR #2
#4	(Tai Ji[Mesh]) OR (Qigong[MeSH]) OR (Exercise Therapy[MeSH]) OR (High-Intensity Interval Training[MeSH]) OR (resistance training[MeSH]) OR (Water Sports[MeSH]) OR (yoga[MeSH])
#5	(((((((((((((((((((((((((((((((((((((((((((((((((((((((((((((((((((((((((((((((((((((Tai Ji[Title/Abstract]) OR (Tai Ji[Title/Abstract])) OR (Tai Chi[Title/Abstract])) OR (Chi, Tai[Title/Abstract])) OR (Tai Chi Chuan[Title/Abstract])) OR (Taiji[Title/Abstract])) OR (Taijiquan[Title/Abstract])) OR (T'ai Chi[Title/Abstract])) OR (Tai Ji Quan[Title/Abstract])) OR (Ji Quan, Tai[Title/Abstract])) OR (Quan, Tai Ji[Title/Abstract])) OR (Tai Ji[MeSH Terms])) OR (baduanjin[Title/Abstract])) OR (wuqinxi[Title/Abstract])) OR (liuzijue[Title/Abstract])) OR (Ch'i Kung[Title/Abstract])) OR (Qi Gong[Title/Abstract])) OR (Qigong[Title/Abstract])) OR (Qigong[MeSH Terms])) OR (daoyin[Title/Abstract])) OR (kinesitherapy[Title/Abstract])) OR (Traditional exercises[Title/Abstract])) OR (Exercise Therapy[MeSH Terms])) OR (Exercise Therapy[Title/Abstract])) OR (Rehabilitation Exercise[Title/Abstract])) OR (Exercise, Rehabilitation[Title/Abstract])) OR (Exercises, Rehabilitation[Title/Abstract])) OR (Rehabilitation Exercises[Title/Abstract])) OR (Therapy, Exercise[Title/Abstract])) OR (Exercise Therapies[Title/Abstract])) OR (Therapies, Exercise[Title/Abstract])) OR (Remedial Exercise[Title/Abstract])) OR (Exercise, Remedial[Title/Abstract])) OR (Exercise, Remedial[Title/Abstract])) OR (Remedial Exercises[Title/Abstract])) OR (Cardiopulmonary rehabilitation[Title/Abstract])) OR (High-Intensity Interval Training[MeSH Terms])) OR (High-Intensity Interval Training[Title/Abstract])) OR (High Intensity Interval Training[Title/Abstract])) OR (High-Intensity Interval Trainings[Title/Abstract])) OR (Interval Training, High-Intensity[Title/Abstract])) OR (Interval Trainings, High-Intensity[Title/Abstract])) OR (Interval Trainings, High-Intensity[Title/Abstract])) OR (Trainings, High-Intensity Interval[Title/Abstract])) OR (High-Intensity Intermittent Exercise[Title/Abstract])) OR (High-Intensity Intermittent Exercise[Title/Abstract])) OR (Exercises, High-Intensity Intermittent[Title/Abstract])) OR (High-Intensity Intermittent Exercises[Title/Abstract])) OR (Sprint Interval Training[Title/Abstract])) OR (Sprint Interval Training[Title/Abstract])) OR (high intensity continuous training[Title/Abstract])) OR (resistance training[Title/Abstract])) OR (resistance training[MeSH Terms])) OR (resistance training[Title/Abstract])) OR (Strength Training[Title/Abstract])) OR (Weight-Lifting Strengthening Program[Title/Abstract])) OR (Weight-Lifting Strengthening Program[Title/Abstract])) OR (Strengthening Programs, Weight-Lifting[Title/Abstract])) OR (Strengthening Program, Weight-Lifting[Title/Abstract])) OR (Weight Lifting Strengthening Program[Title/Abstract])) OR (Weight-Lifting Exercise Program[Title/Abstract])) OR (Exercise Program, Weight-Lifting[Title/Abstract])) OR (Weight Lifting Exercise Program[Title/Abstract])) OR (Weight-Lifting Exercise Programs[Title/Abstract])) OR (Weight-Bearing Strengthening Program[Title/Abstract])) OR (Strengthening Programs, Weight-Bearing[Title/Abstract])) OR (Strengthening Programs, Weight-Bearing[Title/Abstract])) OR (Weight-Bearing Exercise Program[Title/Abstract])) OR (Exercise Programs, Weight-Bearing[Title/Abstract])) OR (Exercise Programs, Weight-Bearing[Title/Abstract])) OR (Water Sports[MeSH Terms])) OR (Water Sports[Title/Abstract])) OR (Water Sports[Title/Abstract])) OR (Water Sports[Title/Abstract])) OR (Kayaking[Title/Abstract])) OR (Kayaking[Title/Abstract])) OR (Canoeing[Title/Abstract])) OR (Water Polo[Title/Abstract])) OR (Surfboarding[Title/Abstract])) OR (Boating[Title/Abstract])) OR (Wave Surfing[Title/Abstract])) OR (Rowing[Title/Abstract])) OR (Skiing, Water[Title/Abstract])) OR (yoga[Title/Abstract])) OR (yoga[MeSH Terms]))
#6	#4 OR #5
#7	#3 AND #6
中国知网数据库检索策略	
#1	高血压+原发性高血压（主题词）
#2	太极拳+太极拳运动+太极拳练习+24式太极拳+陈式太极拳+杨式太极拳（主题词）
#3	运动疗法+运动疗法干预+运动疗法训练+运动疗法康复+‘运动疗法/方法'（主题词）
#4	传统功法+传统功法治疗+传统功法训练（主题词）
#5	八段锦+八段锦锻炼+八段锦运动+八段锦训练+八段锦干预+八段锦练习（主题词）
#6	气功+气功疗法+气功锻炼+中国气功+传统气功+导引（主题词）
#7	运动康复+运动康复训练+运动康复治疗+运动康复干预+运动康复疗法（主题词）
#8	心肺康复+心肺康复训练+心肺康复治疗+心肺康复运动+心肺康复训练指导（主题词）
#9	五禽戏+五禽戏锻炼+五禽戏运动（主题词）
#10	六字诀+六字诀呼吸操+六字诀功法+六字诀训练+六字诀呼吸吐纳功+六字诀养气功（主题词）
#11	HIIT+hiit训练+hiit+hiit干预+‘hiit(高强度间歇训练)’+‘高强度间歇训练(hiit)'（主题词）
#12	高强度持续训练+抗阻训练+水上运动+瑜伽(主题词)
#13	#2 OR #3 OR #4 OR #5 OR #6 OR #7 OR #8 OR #9 OR #10 OR #11 OR #12
#14	#1 AND #13

### 2.2 Inclusion criteria

The inclusion criteria of this study strictly followed the PICOS framework, and the inclusion criteria were as follows: (1) Study type: Published randomized controlled trials (RCTs); (2) Patients: Patients with a clear diagnosis of essential hypertension. There were no specific requirements for the patients' age, gender, etiology, and severity, etc. The diagnostic criteria only needed to meet any one of the domestic and international clinical guidelines for the diagnosis and treatment of hypertension, such as the “Chinese Guidelines for the Prevention and Treatment of Hypertension” (12). The specific diagnostic criteria included SBP ≥ 130 mm Hg (1 mmHg≈0.133 kPa) and/or diastolic blood pressure (DBP) ≥ 80 mmHg, etc.

### 2.3 Intervention measures

This article aims to study the therapeutic effects of different exercise therapies on patients with essential hypertension. Therefore, studies in which different exercise therapies or a combination of exercise therapy and conventional medications were used for intervention in the observation group were included. Exercise therapy can be used alone as a blank control; alternatively, conventional drug treatment can be selected for the control group, and the observation group can receive exercise therapy intervention on the basis of the treatment in the control group.

### 2.4 Efficacy indicators

Main Observation Indicators: Ambulatory Blood Pressure Measurement (ABPM), including 24-h average systolic blood pressure and 24-h average diastolic blood pressure (They will be referred to as SBP and DBP hereinafter).

### 2.5 Exclusion criteria

The exclusion criteria are as follows:(1) Studies that are not randomized controlled trials; (2) Studies involving combined interventions of multiple types of exercises; (3) Articles that are repeatedly published or have been retracted; (4) Literatures for which the full text cannot be downloaded; (5) Literatures with incomplete data of the study subjects and without descriptions of the efficacy indicators; (6) Studies related to secondary hypertension.

### 2.6 Literature screening and data extraction

Two authors (Liu Longcheng and Wang Jiale from Peking Union Medical College) separately completed the screening and extraction of the literatures. Firstly, the literatures that were obviously irrelevant to this article were excluded by reading the titles and abstracts. Then, the literatures that did not meet the conditions of this study were excluded after downloading the full texts and reading them carefully. After the data extraction was completed, the results were compared. In case of any discrepancies, a third researcher was invited to discuss and make a decision together. Finally, the Excel software was used for data extraction. The extracted data included information such as the authors, publication time, study subjects, sample size, intervention measures, treatment course, and efficacy indicators.

### 2.7 Risk of bias assessment

In this article, the bias risk tool described in Cochrane Handbook 5.1.0 was used to assess the methodological quality of the included RCTs. The evaluation mainly covered the following aspects: random allocation method, allocation concealment, blinding of participants, blinding of outcome assessment, data completeness, selective reporting, and other biases. For each item, the risk was respectively determined as “unclear bias risk”, “low bias risk”, or “high bias risk”.

### 2.8 Statistical analysis

The primary outcome of this article is 24-h average blood pressure. To ensure the accuracy of the results, this article calculates the differences between the BP and BP at the study baseline and the endpoint as the research data. The standard deviation of the differences is calculated using the Pearson correlation coefficient related to the blood pressure before and after the intervention. The calculation formula is as follows (13):

$$SD^{2}\mathrm{change}=SD^{2}\mathrm{baseline}\;+\;SD^{2}\mathrm{final}-(2\times \mathrm{Corr}\;\times \;\mathrm{SDbaseline}\;\times \;\mathrm{SDfinal})$$

In this article, software such as Stata 15.0 and Rev Man 5.4 were used for data analysis in the Network analysis. For the measurement data in this article, the weighted mean difference (WMD) and its 95% confidence interval (CI) were used for analysis. When there are loops in the Network map, the node—splitting method is used for the inconsistency test. When *P* > 0.05, it is considered that there is no heterogeneity. The Stata 15.0 software is used to conduct the cumulative ranking curve (SUCRA) curve ranking to obtain the optimal intervention measure. Meta-regression and sensitivity analysis were performed using Stata 15.0. The Stata 15.0 software is used to conduct Begg's test. If *P* > 0.05, it proves that there is no publication bias.

## 3 Results

### 3.1 Literature screening

**Figure 1:**
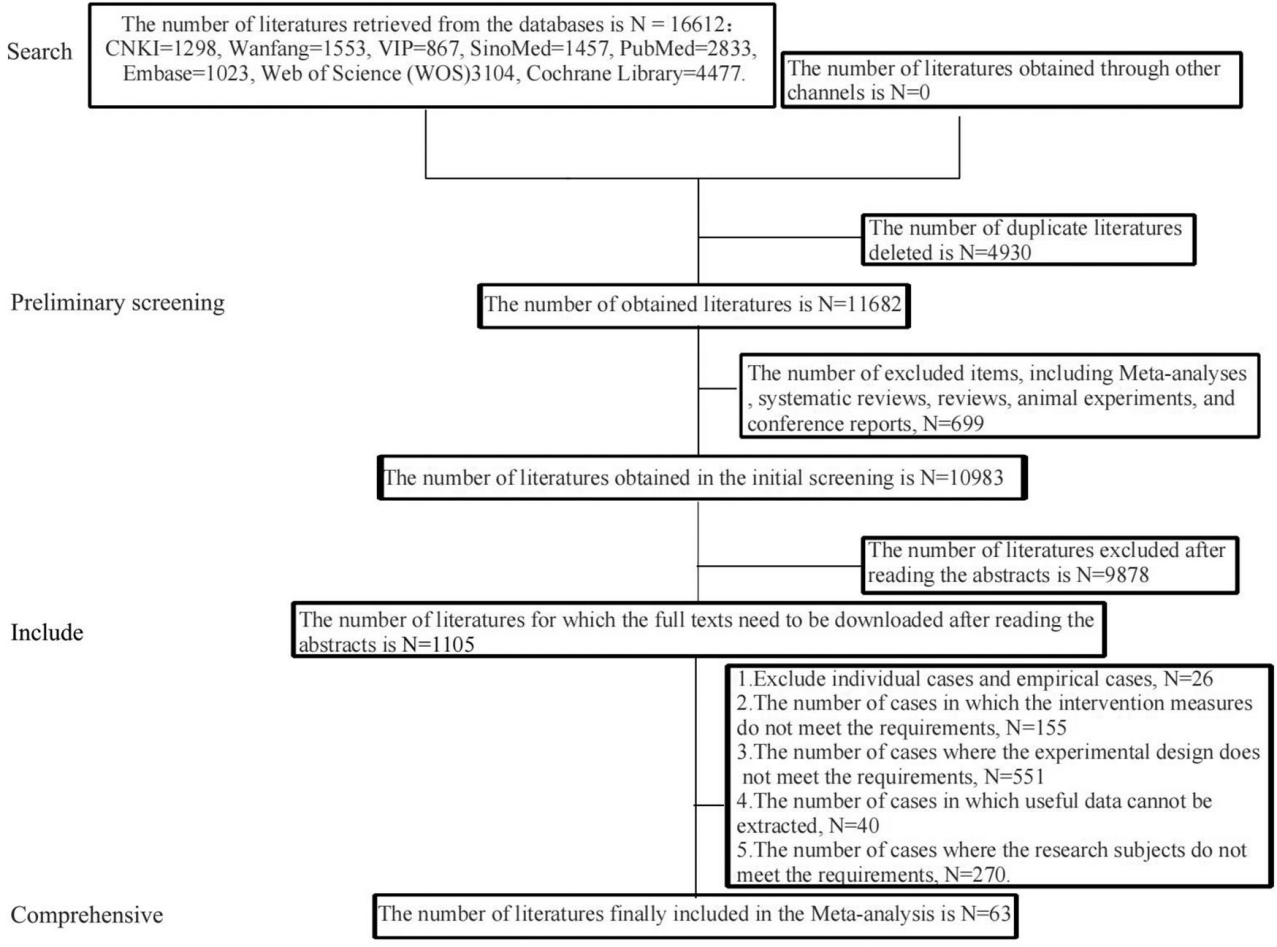
The process of literature inclusion and exclusion.

A total of 16,612 literatures were initially retrieved. After removing the duplicate and incorrect literatures, 11,682 literatures remained. Then, 699 literatures, including Meta-analyses, systematic reviews, animal experiments, and conference reports, were excluded. After reading the titles and abstracts, 9,878 literatures were excluded. Finally, 63 literatures were included after reading the full texts. The intervention measures involved a total of 12 types of exercise therapies, namely TC, BDJ, WQX, low-intensity aerobic exercise(LIAE), moderate-intensity aerobic exercise(MIAE), Liuzijue(LZJ), Isometric exercise(IE), Isometric grip strength(IGS), HIIT, Qigong(QG), Resistance training(RT), and Yoga. The process of inclusion and exclusion is shown in Figure 1.

### 3.2 Basic characteristics of the included literature

A total of 63 RCTs (14–76) were finally included, among which (14–27, 29–67, 69–70, 72–76) were two-arm trials and 3 trials (28, 68, 71) were three-arm trials. The total sample size was 5,663 cases, including 2,826 cases in the experimental group and 2,837 cases in the control group. The shortest course of treatment in the observation group was 6 weeks, and the longest was 12 months. The most common practice frequency was 1–2 times a day, and the duration of each practice mostly remained around 2 h. The baseline data of the patients were all comparable. The basic characteristics of the included literature are shown in Table 2.

**Table 2:** Basic characteristics of the literature.

Number	Included literature	Samplesize/cases		Male/female/example		Average age/years		Intervening measure		Course of treatment	Outcome indicator	Weekly intervention time
		T	C	T	C	T	C	T	C			
1	Chunling and Zhang (14)	30	30	–	–	–	–	BDJ + CON	CON	2M	BP	4–5 h
2	Qinghua et al. (15)	15	15	–	–	54 ± 6	53 ± 8	TC + CON	CON	3M	BP	3–5 h
3	Huan (16)	30	30	17/13	18/12	38.07 ± 8.09	37.63 ± 9.09	TC + CT	CT	2M	BP	2–3 h
4	Xiaobin and Luping (17)	50	50	20/30	22/28	66.6 ± 4.5	66.4 ± 4.2	TC + CT	CT	3M	BP	2–3 h
5	Lijuan et al. (18)	36	37	19/17	14/23	66.33 ± 4.74	67.51 ± 4.09	TC + CT	CT	3M	BP	3 h
6	Yujie (19)	40	40	25/15	22/18	67.98 ± 4.87	67.14 ± 5.01	BDJ + CT	CT	6M	BP	3–4 h
7	Fang and He (20)	27	28	14/13	12/16	61.26 ± 3.74	62.03 ± 3.51	BDJ + CT	CT	3M	BP	7 h
8	Hongyu et al. (21)	30	30	17/13	16/14	68.1 ± 10.1	70.5 ± 10.2	BDJ + CT	CT	3M	BP	–
9	Hui and Yana (22)	40	40	25/15	23/17	59 ± 6	60 ± 5	BDJ + CT	CT	6M	BP	4–5 h
10	Chen (23)	112	114	68/44	70/44	60.89 ± 9.70	61.33 ± 8.23	BDJ + CON	CON	12M	BP	4–5 h
11	Liwei et al. (24)	30	30	13/14	16/12	69.23 ± 3.72	70.06 ± 3.51	BDJ + CT	CT	3M	BP	2–3 h
12	Yunhua et al. (25)	30	30	10/20	18/12	54.8 ± 7.6	55.7 ± 8.8	BDJ + CT	CT	6M	BP	2–3 h
13	Zhanmei et al. (26)	77	77		–	–	–	BDJ + CT	CT	3M	BP	4–5 h
14	Yakang (27)	42	42	24/18	22/20	6O.2 ± 4.6	60.5 + 4.9	BDJ + CON	CON	3M	BP	4–5 h
15	Jingyuan (28)	32	32	16/16	17/15	71.5 ± 9.5	73.5 ± 10.5	MIAE + CT	CT	3M	BP	3 h
		32	32	18/14	17/15	72 ± 9	73.5 ± 10.5	RT + CT	CT	3M	BP	3 h
16	Chunli (29)	50	50	28/22	26/24	68.4 ± 2.3	69.7 ± 2.8	LIAE + CT	CT	6M	BP	1–2 h
17	Peiqin (30)	30	30	18/12	19/11	71.65 ± 2.73	71.59 ± 2.68	LIAE + CON	CON	6M	BP	1–2 h
18	Yingxian (31)	48	48	29/19	27/21	68.72 ± 4.03	68.43 ± 3.96	LIAE + CON	CON	1M	BP	3–5 h
19	Linghua et al. (32)	42	42	22/20	21/21	52.52 ± 2.47	52.25 ± 2.30	HIIT + CON	CON	3M	BP	5–7 h
20	Lei et al. (33)	50	50	32/28	30/30	–	–	MIAE + CT	CT	3M	BP	3–5 h
21	Lixun and Jianquan (34)	55	55	25/30	24/31	64.10 ± 7.03	64.21 ± 6.12	TC + CT	CT	3M	BP	4–5 h
22	Qiu (35)	58	58		–	–	–	BDJ + CT	CT	6M	BP	4–5 h
23	Jian (36)	18	18	11/7	13/5	49.6 ± 7.1	46.8 ± 6.4	RT + CT	CT	4M	BP	5–7 h
24	Na (37)	75	18	40/35	39/36	55.1 ± 3.1	54.3 ± 2.8	RT + CON	CON	3M	BP	3–5 h
25	Xue (38)	42	42	22/20	23/19	68.51 ± 2.97	69.24 ± 2.45	BDJ + CON	CON	3M	BP	2–3 h
26	Xiaoduo et al. (39)	50	50	15/35	17/33	57.5 ± 10.16	58 ± 10.03	LZJ + CT	CT	3M	BP	5–7 h
27	Sun et al. (40)	136	130		–	–	–	BDJ + CON	CON	12M	BP	4–5 h
28	Haolei and Jiajia (41)	27	27		–	–	–	BDJ + CT	CT	6M	BP	4–5 h
29	Yamei et al. (42)	40	40	29/19	22/18	66.98 ± 5.48	67.02 ± 5.46	BDJ + CT	CT	3M	BP	5–7 h
30	Hua (43)	44	40	24/20	21/19	44.74 ± 12.10	44.86土13.05	BDJ + CT	CT	6M	BP	4–5 h
31	Tao et al. (44)	35	35	18/17	19/16	62.4 ± 2.4	63.1 ± 2.1	BDJ + CT	CT	6M	BP	5–7 h
32	Wen et al. (45)	30	30		–	–	–	WQX + CT	CT	6M	BP	2–3 h
33	Huihui (46)	43	43	21/22	20/23	46	45	WQX + CT	CT	3M	BP	3–4 h
34	Jiangqin and Yujie (47)	50	50	28/22	29/21	72.35 ± 2.31	73.02 ± 2.35	TC + CT	CT	3M	BP	5–7 h
35	Yong et al. (48)	27	31		–	39 ± 3.32	42 ± 2.86	BDJ + CON	CON	3M	BP	1–2 h
36	Yi (49)	60	60	39/21	38/22	69.40 ± 6.33	69.33 ± 5.12	MIAE + CT	CT	6M	BP	3–5 h
37	Yanli (50)	70	66	41/29	39/27	53	53.5	MIAE + CT	CT	6M	BP	5–7 h
38	Tianming (51)	36	40	19/17	18/22	63.9 ± 3.8	64.5 ± 3.2	MIAE + CT	CT	6M	BP	5–7 h
39	Suo Mengmeng (52)	52	52	32/20	31/21	53.1士5.1	52.5士4.9	MIAE + CT	CT	-	BP	5 h
40	Xiaofang and Yongxin (53)	36	39	24/12	22/17	39—68	41—72	MIAE + CT	CT	6M	BP	5 h
41	Minfang (54)	36	36	20/16	21/15	52.8 ± 15.8	53.8 ± 14.7	MIAE + CT	CT	1M	BP	1–2 h
42	Xiuling (55)	75	36	37/38	39/36	58.7 ± 10.2	59.1 ± 10.5	MIAE + CT	CT	8W	BP	2 h
43	Qing and Ping (56)	33	36		–	–	–	MIAE + CT	CT	7W	BP	2 h
44	Yulong (57)	34 36	36	20/14	21/13	68.5 ± 2.4	68.6 ± 2.3	MIAE + CT	CT	7W	BP	2 h
45	Chan et al. (58)	82	82	32/50	38/44	64.70 ± 7.59	65.13 ± 10.22	TC + CON	CON	3M	BP	2 h
46	Hou et al. (59)	44	43	15/29	15/28	68.84 ± 4,45	67.44 ± 4.94	MIAE + CON	CON	4M	BP	3 h
47	Maruf et al. (60)	60	60	13/47	22/38	50.8 ± 8.31	54.75 ± 8.56	MIAE + CT	CT	3M	BP	4–5 h
48	Ping and Zhong (61)	60	60	32/28	34/26	62.74 ± 3.06	62.15 ± 3.66	TC + CT	CT	3M	BP	3 h
49	Kaiyong (62)	30	30	25/5	23/7	63.58 ± 4.20	63.46 ± 4.13	MIAE + CT	CT	3M	BP	2 h
50	Cooper et al. (63)	48	42	39/9	33/9	46.2 ± 9.7	49.4 ± 8.9	MIAE + CON	CON	6W	BP	2–3 h
51	Cramer et al. (64)	25	25		–	–	–	Yoga + CON	CON	7M	BP	1.5 h
52	Cohen et al. (65)	46	32	23/23	16/16	48.2 ± 1.6	48.3 ± 2.4	Yoga + CON	CON	3M	BP	–
53	Anjana et al. (66)	34	31	15/19	14/17	49.13 ± 8.106	43.90 ± 9.24	Yoga + CT	CT	2M	BP	4–5 h
54	Molmen-Hansen et al. (67)	28	29	16/12	17/12	53.6 ± 6.5	51.3 ± 9.2	MIAE + CON	CON	3M	BP	3 h
55	Gorostegi-Anduaga et al. (68)	42	45	28/14	30/15	54.7 ± 7.6	53.1 ± 8.3	MIAE + CT	CT	4M	BP	2–3 h
		44	45	32/12	30/15	53.5 ± 9.1	53.1 ± 8.3	HIIT + CT	CT	4M	BP	2–3 h
56	Chen (69)	30	30		–	–	–	QG + CON	CON	4M	BP	4–5 h
57	Xiao et al. (70)	24			–	–	–	BDJ + CON	CON	6M	BP	4–5 h
58	Cohen et al. (71)	28	22		–	–	–	IGS + CON	CON	3M	BP	–
		27	22		–	–	–	IE + CON	CON	3M	BP	–
59	Wolff et al. (72)	96	95	44/52	48/47	64.7 ± 9.2	64.8 ± 7.6	Yoga + CT	CT	3M	BP	3–4 h
60	Punia et al. (73)	20	20	–	–	–	–	IGS + CT	CT	8W	BP	1–2 h
61	Izadi et al. (74)	15	15	7/7	9/6	–	–	HIIT + CT	CT	6W	BP	3–4 h
62	Shou et al. (75)	98	100	48/50	55/45	–	–	TC + CON	CON	3M	BP	5–7 h
63	McCaffrey et al. (76)	27	27	10/17	9/18	56.7	56.2	Yoga + CON	CON	8W	BP	2–3 h

T, experimental group; C, control group—not reported; CT: standard medication; CON: untreated control; BP:24-h average blood pressure; TC, TiChi; BDJ: Baduanjin; WQX, Wuqinxi; LIAE, low-intensity aerobic training; MIAE, moderate-intensity aerobic exercise; LZJ, Liuzijue; IE, isometric exercise training; IGS, isometric grip strength training; HIIT, high-intensity interval training; QG, Qigong; RT, resistance training; BP, blood pressure; W, weeks; M, months.

### 3.3 Assessment of bias risk

**Figure 2:**
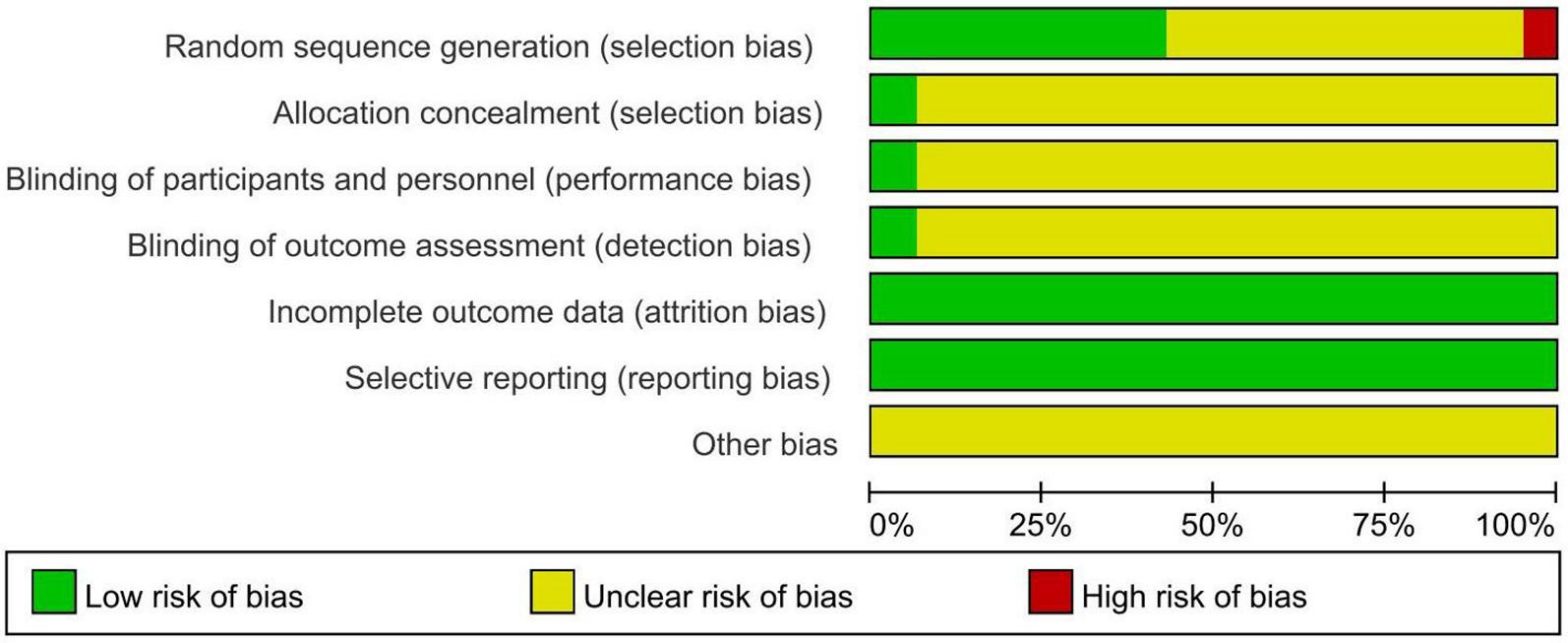
Risk of bias assessment of included studies.

Using the RoB scale recommended by Cochrane as the evaluation tool, and the risk bias graph was drawn by the Revman software. A total of 38 RCTs (19, 21–23, 26, 28–30, 36, 37, 40, 41, 43, 45, 46, 49, 50, 52, 53, 55, 61, 63, 65–71, 73–76) mentioned randomization, but did not describe the specific method, and were rated as “unclear bias risk”; 23 RCTs (14, 16–18, 20, 25, 27, 31–34, 37–39, 42, 44, 47, 48, 54, 56, 57, 62, 64) used the random number table method, and 4 RCTs (58–60, 72) used computer randomization, all of which were rated as “low bias risk”. 2 RCTs (24, 51) used the odd-even method and convenience sampling method, and were rated as “high bias risk”. All the literatures did not mention allocation concealment and other sources of bias, and were all rated as “unclear bias risk”. In terms of the implementation of blinding, 4 RCTs (64, 66, 72, 73) adopted double blinding and were rated as “low bias risk”; the rest did not mention blinding and were all rated as “unclear bias risk”. In terms of data integrity assessment, most of the articles had no dropped cases and were rated as “low bias risk”, and a small number of articles that mentioned dropped cases all gave reasons and were all rated as “unclear bias risk”. All the articles had no selective reporting bias and were all rated as “low bias risk”, as shown in Figure 2.

### 3.4 Evidence network

**Figure 3:**
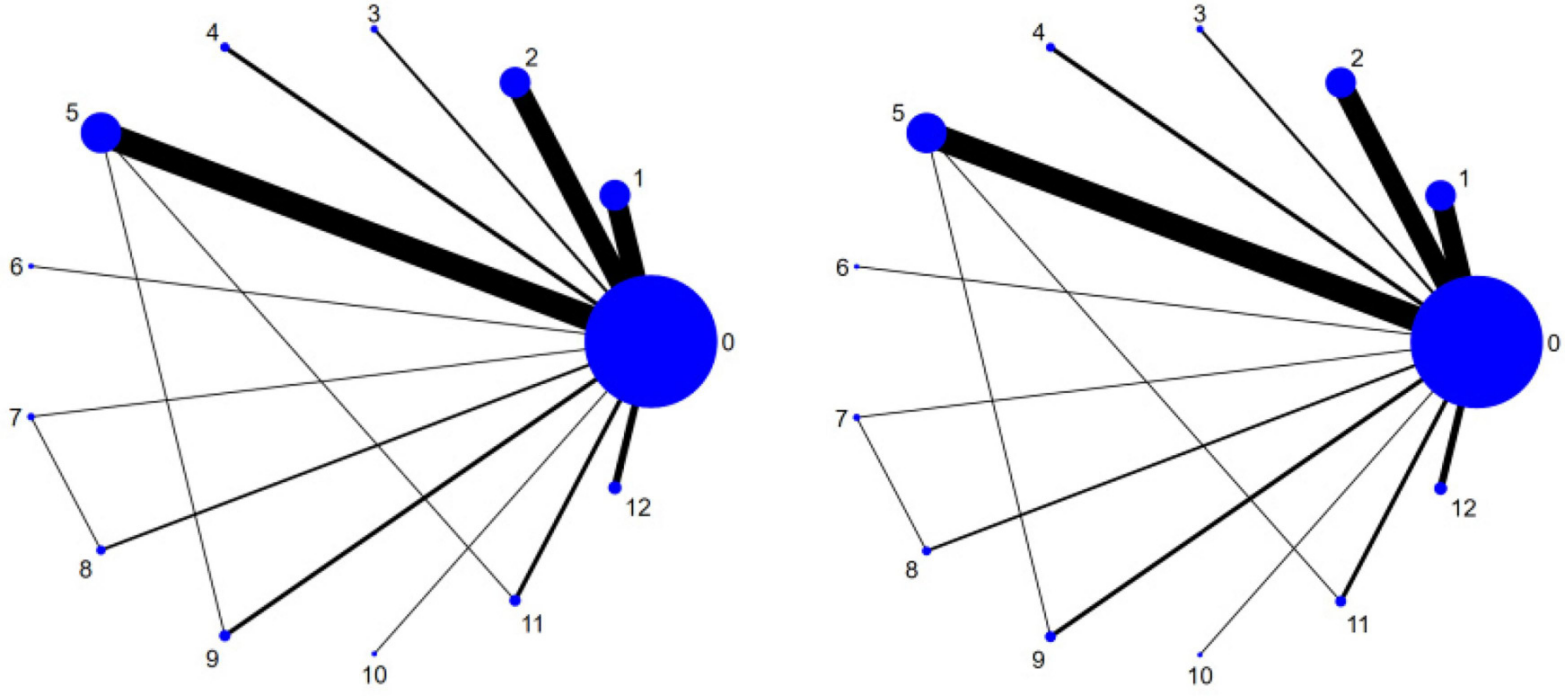
Network map.

**Figure 4:**
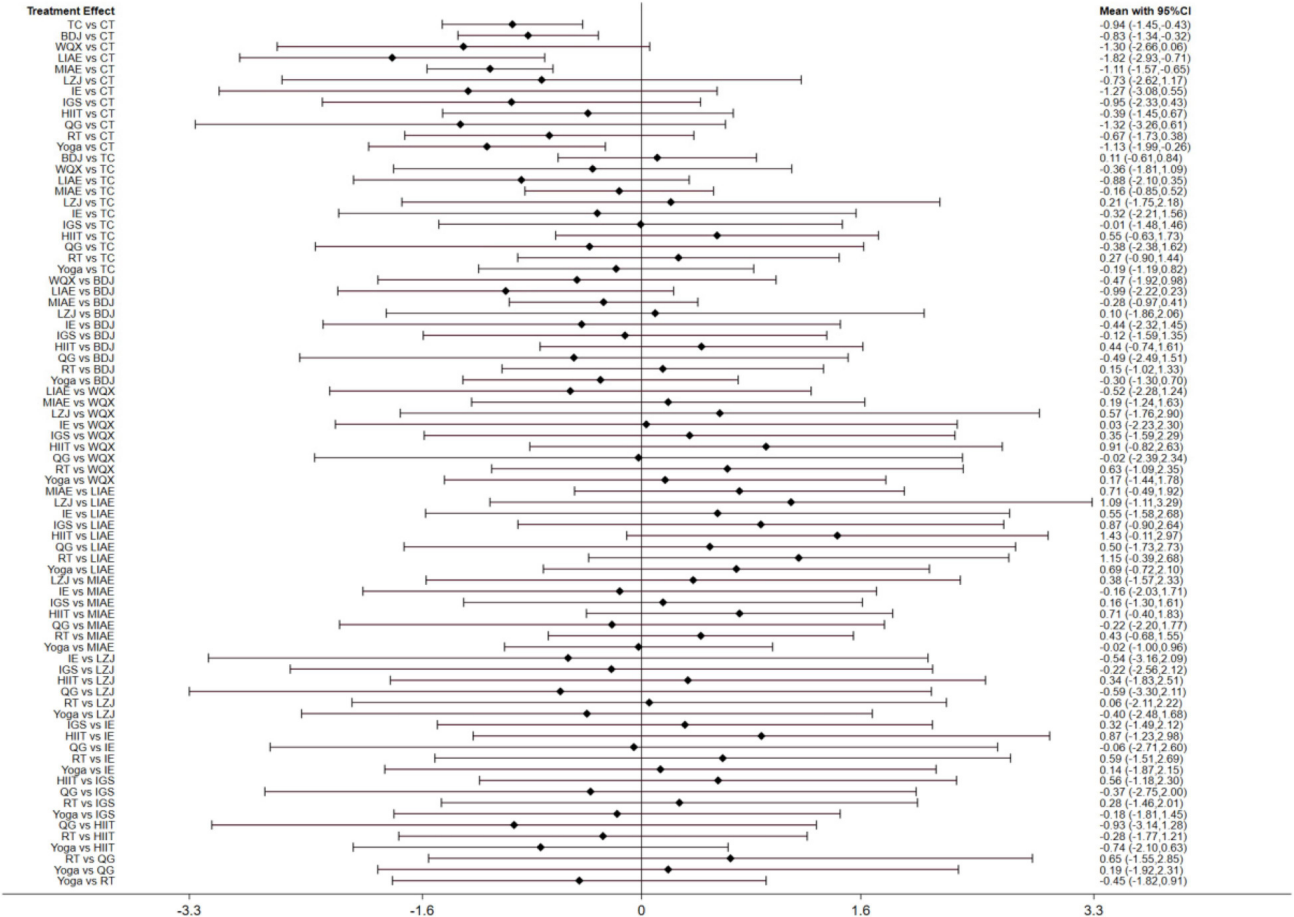
SBP forest plot. The left image is the Network Map of SBP, and the right image is the Network Map of DBP. The corresponding labels are: (0) CT; (1) TC; (2) BDJ; (3) WQX; (4) LIAE; (5) MIAE; (6) LZJ; (7) IE; (8) IGS; (9) HIIT; (10) QG; (11) RT; (12) Yoga.

**Figure 5:**
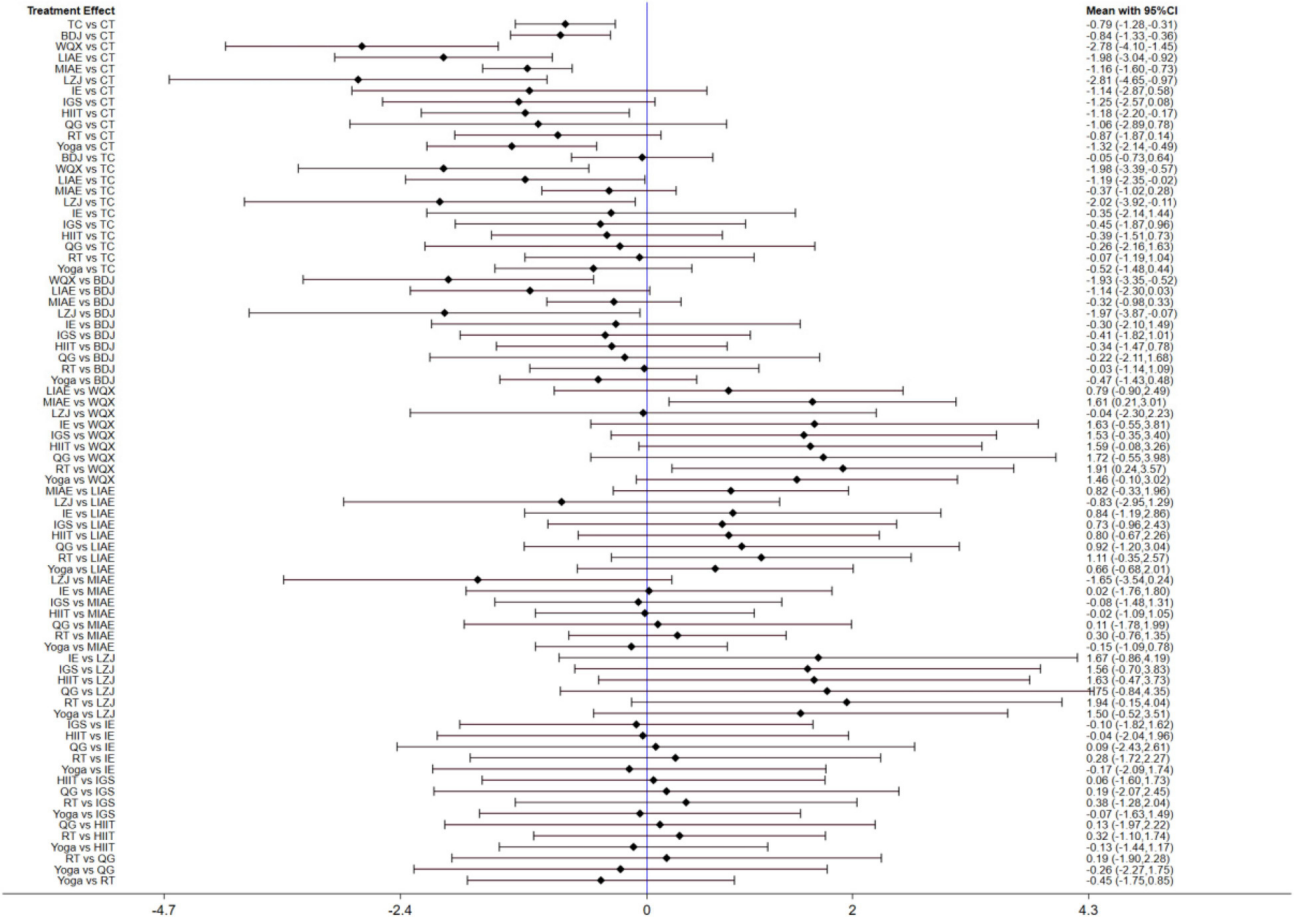
DBP forest plot.

A total of 63 studies (14–76) and 12 intervention measures were included in the network meta-analysis. The network diagram is shown as follows. The inconsistency test indicates good consistency (*p* > 0.05), and the consistency test model was adopted for statistical analysis. In the figure, the circles represent the intervention measures, and the diameter is positively correlated with the included sample size; the connecting lines represent that there is a direct comparison between the two intervention measures. The thicker the straight line between the two points, the larger the number of relevant RCTs, as shown in Figure 3. The visualization forest plot is shown in Figures 4, 5.

### 3.5 Network meta-analysis

#### 3.5.1 SBP

A total of 63 studies (14–76) reported the results of SBP. Through network meta-analysis, CT + TC [weighted mean difference (WMD) = −0.94, 95%CI (−1.45, −0.43)], CT + BDJ [WMD = −0.83, 95% CI(−1.34, −0.32)], CT + LIAE [WMD = −1.82, 95% CI(−2.93, −0.71)], CT + MIAE [WMD = −1.11, 95% CI(−1.57, −0.65)], CT + Yoga [WMD = −1.13, 95% CI(−1.99, −0.26)] were significantly superior to the control group which only used antihypertensive drugs or nursing measures to reduce SBP. However, as shown in Table 3, there was no statistical significance among other intervention measures.

**Table 3:** Shows the comparison results of different types of exercise therapies in reducing SBP.

Intervention measures	MD [95%CI]												
	CON	TC	BDJ	WQX	LIAE	MIAE	LZJ	IE	IGS	HIIT	QG	RT	Yoga
CON	0												
TC	**−0.94 [−1.45, −0.43]**	0											
BDJ	**−0.83 [−1.34, −0.32]**	0.11 [−0.61, 0.84]	0										
WQX	−1.3[−2.66, 0.06]	−0.36 [−1.81, 1.09]	−0.47 [−1.92, 0.98]	0									
LIAE	**−1.82 [−2.93, −0.71]**	−0.88 [−2.1,0.35]	−0.99 [−2.22, 0.23]	−0.52 [−2.28, 1.24]	0								
MIAE	**−1.11 [−1.57 , −0.65]**	−0.16 [−0.85, 0.52]	−0.28 [−0.97, 0.41]	0.19 [−1.24, 1.63]	0.71 [−0.49, 1.92]	0							
LZJ	−0.73 [−2.62, 1.17]	0.21 [−1.75, 2.18]	0.1 [−1.86, 2.06]	0.57 [−1.76, 2.9]	1.09 [−1.11, 3.29]	0.38 [−1.57, 2.33]	0						
IE	−1.27 [−3.08, 0.55]	−0.32 [−2.21, 1.56]	−0.44 [−2.32, 1.45]	0.03 [−2.23, 2.3]	0.55 [−1.58, 2.68]	−0.16 [−2.03, 1.71]	−0.54 [−3.16,2.09]	0					
IGS	−0.95 [−2.33, 0.43]	−0.01 [−1.48, 1.46]	−0.12 [−1.59, 1.35]	0.35[−1.59, 2.29]	0.87 [−0.9, 2.64]	0.16 [−1.3, 1.61]	−0.22 [−2.56, 2.12]	0.32 [−1.49, 2.12]	0				
HIIT	−0.39 [−1.45, 0.67]	0.55 [−0.63, 1.73]	0.44 [−0.74, 1.61]	0.91 [−0.82, 2.63]	1.43 [−0.11 ,2.97]	0.71 [−0.4, 1.83]	0.34 [−1.83, 2.51]	0.87 [−1.23, 2.98]	0.56 [−1.18, 2.3]	0			
QG	−1.32 [−3.26, 0.61]	−0.38 [−2.38, 1.62]	−0.49 [−2.49, 1.51]	−0.02 [−2.39, 2.34]	0.5 [−1.73, 2.73]	−0.22 [−2.2, 1.77]	−0.59 [−3.3, 2.11]	−0.06 [−2.71, 2.6]	−0.37 [−2.75, 2]	−0.93 [−3.14, 1.28]	0		
RT	−0.67 [−1.73, 0.38]	0.27 [−0.9, 1.44]	0.15 [−1.02, 1.33]	0.63 [−1.09, 2.35]	1.15 [−0.39, 2.68]	0.43 [−0.68, 1.55]	0.06 [−2.11, 2.22]	0.59 [−1.51, 2.69]	0.28 [−1.46, 2.01]	−0.28 [−1 .77, 1.21]	0.65 [−1.55, 2.85]	0	
Yoga	**−1.13 [−1.99, −0.26]**	−0.19 [−1.19, 0.82]	−0.3 [−1.3, 0.7]	0.17 [−1.44, 1.78]	0.69 [−0.72, 2.1]	−0.02 [−1, 0.96]	−0.4 [−2.48, 1.68]	0.14 [−1.87, 2.15]	−0.18 [−1.81, 1.45]	−0.74 [−2.1, 0.63]	0.19 [−1.92, 2.31]	−0.45 [−1.82, 0.91]	0

The results in bold black font indicate that the pairwise comparison results are statistically significant.

#### 3.5.2 DBP

A total of 63 studies (14–76) reported the results of DBP. Through network meta-analysis, compared with traditional antihypertensive drugs and nursing education, etc., CT + TC [WMD = −0.79, 95%CI (−1.28, −0.31)], CT + BDJ [WMD = −0.84, 95% CI(−1.33, −0.36)], CT + WQX [WMD = −2.78, 95% CI(−4.1, −1.45)], CT + LIAE [WMD = −1.98, 95% CI(−3.04, −0.92)], CT + MIAE [WMD = −1.16, 95% CI(−1.6, −0.73)], CT + HIIT [WMD = −1.18, 95% CI(−2.2, −0.17)], CT + Yoga [WMD = −1.32, 95% CI(−2.14, −0.49)] could more significantly reduce DBP. As shown in Table 4, there was no statistical difference between other intervention methods and the control group.

**Table 4:** Shows the comparison results of different types of exercise therapies in reducing DBP.

Intervention measures	MD[95%CI]												
	CON	TC	BDJ	WQX	LIAE	MIAE	LZJ	IE	IGS	HIIT	QG	RT	Yoga
CON	0												
TC	**−0.79 [−1.28, −0.31 ]**	0											
BDJ	**−0.84 [−1.33, −0.36]**	−0.05 [−0.73, 0.64]	0										
WQX	**−2.78 [−4.1, −1.45]**	**−1.98 [−3.39, −0.57]**	**−1.93 [−3.35, −0.52]**	0									
LIAE	**−1.98 [−3.04, −0.92]**	**−1.19 [−2.35, −0.02]**	−1.14 [−2.3, 0.03]	0.79 [−0.9, 2.49]	0								
MIAE	**−1.16 [−1.6, −0.73]**	−0.37 [−1.02, 0.28]	−0.32 [−0.98, 0.33]	**1.61 [0.21, 3.01]**	0.82 [−0.33, 1.96]	0							
LZJ	**−2.81[−4.65, −0.97]**	**−2.02 [−3.92, −0.11]**	**−1.97 [−3.87, −0.07]**	−0.04 [−2.3, 2.23]	−0.83 [−2.95, 1.29]	−1.65 [−3.54, 0.24]	0						
IE	−1.14 [−2.87, 0.58]	−0.35 [−2.14, 1.44]	−0.3 [−2.1, 1.49]	1.63 [−0.55, 3.81]	0.84 [−1.19, 2.86]	0.02 [−1.76, 1.8]	1.67 [−0.86, 4.19]	0					
IGS	−1.25 [−2.57, 0.08]	−0.45 [−1.87, 0.96]	−0.41 [−1.82, 1.01]	1.53[−0.35, 3.4]	0.73 [−0.96, 2.43]	−0.08 [−1.48, 1.31]	1.56 [−0.7, 3.83]	−0.1 [−1.82, 1.62]	0				
HIIT	**−1.18 [−2.2, −0.17]**	−0.39 [−1.51, 0.73]	−0.34 [−1.47, 0.78]	1.59 [−0.08, 3.26]	0.8 [−0.67, 2.26]	−0.02 [−1.09, 1.05]	1.63 [−0.47, 3.73]	−0.04 [−2.04, 1.96]	0.06 [−1.6, 1.73]	0			
QG	−1.06 [−2.89, 0.78]	−0.26 [−2.16, 1.63]	−0.22 [−2.11, 1.68]	1.72 [−0.55, 3.98]	0.92 [−1.2, 3.04]	0.11 [−1.78, 1.99]	1.75 [−0.84, 4.35]	0.09 [−2.43, 2.61]	0.19 [−2.07, 2.45]	0.13 [−1.97, 2.22]	0		
RT	−0.87 [−1.87, 0.14]	−0.07 [−1.19, 1.04]	−0.03 [−1.14, 1.09]	**1.91 [0.24, 3.57]**	1.11 [−0.35, 2.57]	0.3 [−0.76, 1.35]	1.94 [−0.15, 4.04]	0.28 [−1.72, 2.27]	0.38 [−1.28, 2.04]	0.32 [−1.1, 1.74]	0.19 [−1.9, 2.28]	0	
Yoga	**−1.32[−2.14, −0.49]**	−0.52 [−1.48, 0.44]	−0.47 [−1.43, 0.48]	1.46 [−0.1, 3.02]	0.66 [−0.68, 2.01]	−0.15 [−1.09, 0.78]	1.5 [−0.52,3.51]	−0.17 [−2.09, 1.74]	−0.07 [−1.63, 1.49]	−0.13 [−1.44, 1.17]	−0.26 [−2.27, 1.75]	−0.45 [−1.75, 0.85]	0

The results in bold black font indicate that the pairwise comparison results are statistically significant.

#### 3.5.3 SUCRA ranking

**Figure 6:**
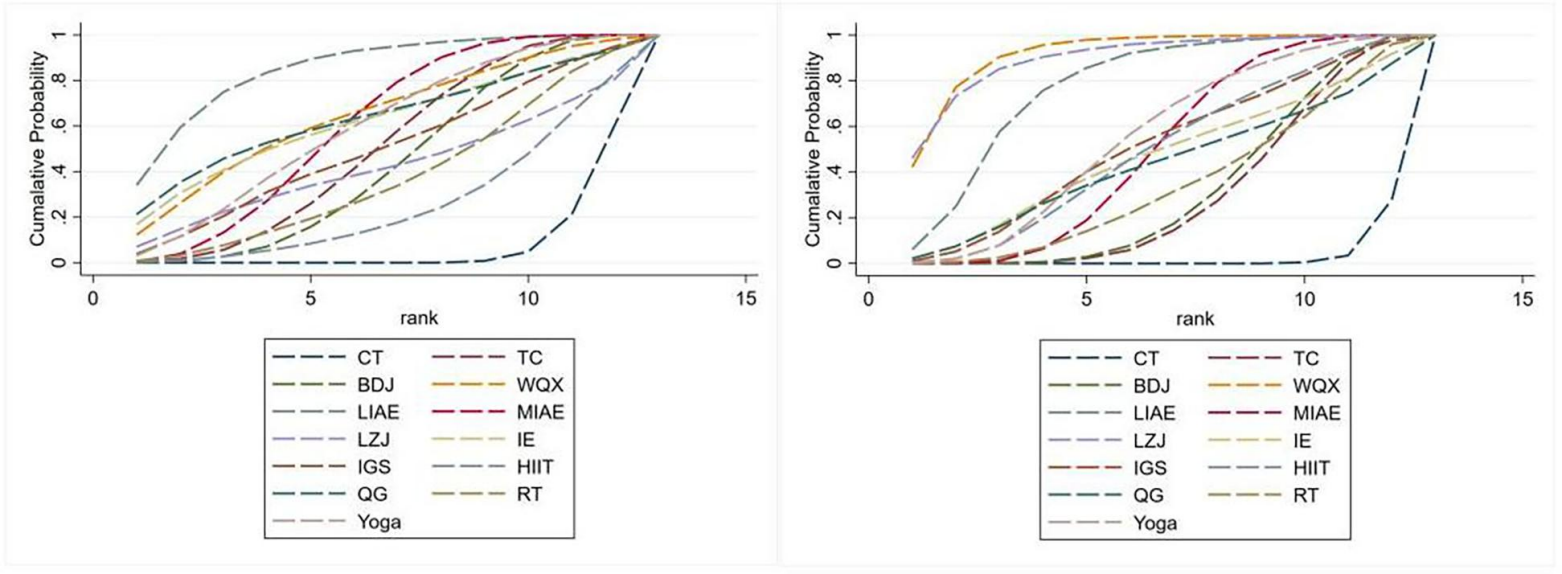
SUCRA ranking.

We ranked different exercise therapies according to the results calculated by SUCRA. The SUCRA probability ranking results of different intervention measures in reducing SBP showed that in terms of reducing SBP, LIAE + CT (SUCRA = 85.3%)>MIAE + CT(SUCRA = 60%)>Yoga + CT(SUCRA = 59.6%)>TC + CT(SUCRA = 50.1%)>BDJ + CT(SUCRA = 43.4%)>CT (SUCRA = 7.3%), and other intervention measures had no statistical significance; in terms of reducing DBP, WQX + CT(SUCRA = 91.8%)>LZJ + CT(SUCRA = 89.8%)>LIAE + CT(SUCRA = 77.6%)> Yoga + CT(SUCRA = 54.8%)>MIAE + CT(SUCRA = 49.3%)>HIIT + CT (SUCRA = 48.8%)>BDJ + CT(SUCRA = 31.4%)>TC + CT(SUCRA = 29.4%)>CT (SUCRA = 2.7%), and other intervention measures had no statistical significance See Figure 6 for details.

### 3.6 Meta-regression

Considering the differences in the types of control groups among the included studies, we conducted a meta-regression analysis to assess their potential impact on the blood pressure-lowering effect. The results of the meta-regression analysis showed that different control types did not affect the systolic blood pressure-lowering effect (*P* = 0.892 > 0.05); nor did different control types affect the diastolic blood pressure-lowering effect (*P* = 0.675 > 0.05).

### 3.7 Sensitivity analysis

To explore the source of heterogeneity, we conducted a sensitivity analysis by excluding individual studies one by one, and the results are detailed in Supplementary Material 1. Consistent with the results of the initial analysis, excluding a single study had little impact on the combined results, which indicates that the combined effect size results of this study are more stable.

### 3.8 Inconsistency test

Both the network diagrams of SBP and DBP have closed loops. Therefore, a local inconsistency test is required. The results showed that there was no significant difference between the direct and indirect analyses of the network analysis for both SBP and DBP (*P* > 0.05), indicating good consistency.

### 3.9 Publication bias

**Figure 7:**
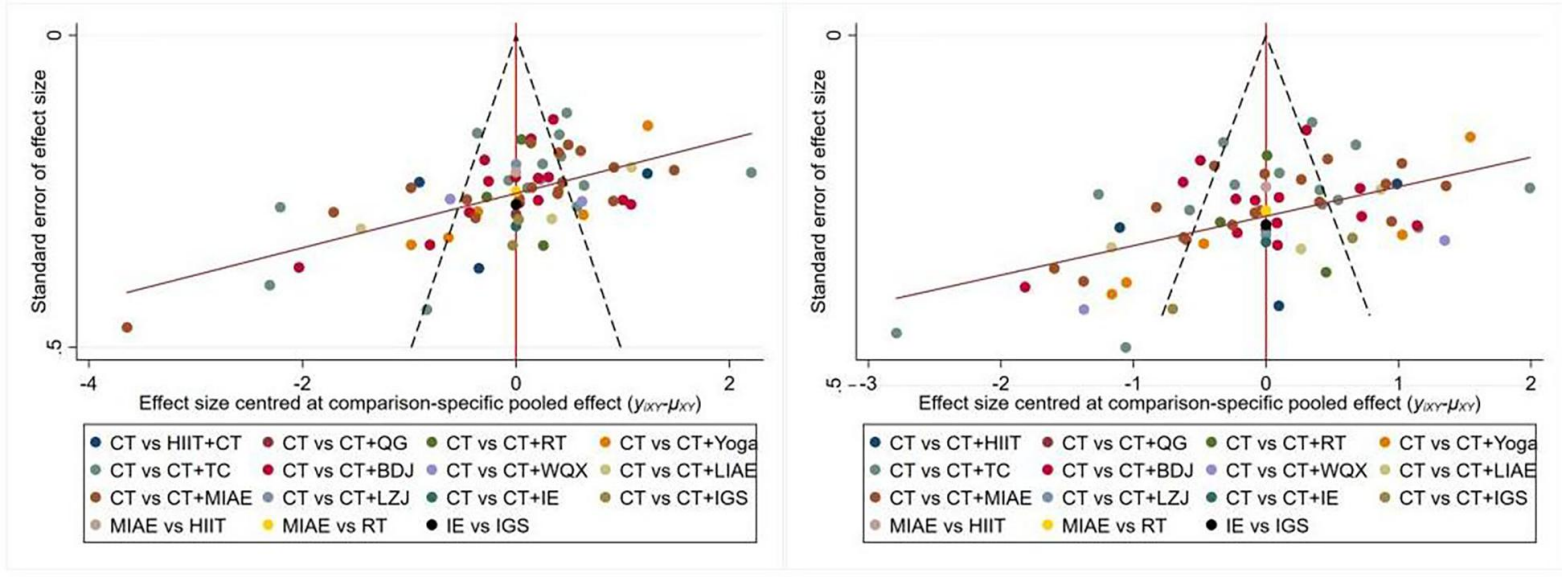
Bias-specific pooled effect.

The Stata15.0 software was used to draw the comparison-corrected funnel plots for different outcome indicators, as shown in Figure 7. The results showed that the scatter distributions of each comparison group in the figure were different. Most of the scatter points were distributed on both sides of the vertical line of X = 0, but there were differences in the degree of symmetry, and many literatures deviated from the axis. The results of Egger's test showed that (for SBP, *P* < 0.05; for DBP, *P* < 0.05), indicating that there was a certain publication bias in the included literatures.

## 4 Discussion

### 4.1 Summary

In recent years, numerous epidemiological studies have linked hypertension to higher risks of stroke, ischemic heart disease, and heart failure (13). Meanwhile, hypertension prevalence in China has risen annually, with a more notable upward trend among young people and in rural areas. Though awareness of hypertension has improved among Chinese patients, it remains relatively low overall (12). Studies confirm active exercise intervention effectively maintains and lowers blood pressure. Thus, the latest hypertension guidelines widely recommend exercise therapies—primarily aerobic and resistance exercise—for hypertension prevention and treatment (7, 12). Specifically, aerobic exercise can reduce adult blood pressure by 5–7 mmHg, while resistance training has an even better blood pressure-lowering effect (10). Aerobic exercise of all intensities works, but many struggle to stick with it long-term due to poor baseline fitness, overly long training sessions, or environmental factors (77).

Currently, traditional exercises (Tai Chi, Baduanjin, Wuqinxi, Liuzijue) are widely used in hypertension management, with clinical studies verifying their good blood pressure-lowering effects (78). This article compares the efficacy of different exercise therapies (aerobic, resistance, yoga, traditional exercises) in hypertension patients. It included 63 RCTs (total sample: 5,663 cases; 2,826 in experimental group, 2,837 in control group) and evaluated 12 exercise therapies. Results showed combinations like TC + CT, BDJ + CT, LIAE + CT, MIAE + CT, WQX + CT, LZJ + CT, HIIT + CT, and Yoga + CT had the best effects—LIAE + CT was most effective for SBP reduction, and WQX + CT for DBP reduction.

In this article, various types of exercise therapies are mentioned. Among them, common types such as low-intensity aerobic exercise are characterized by low exercise intensity, with the heart rate being approximately 60%–70% of the maximum heart rate. It mainly relies on aerobic metabolism for energy supply, has a long duration, and is highly safe. Moderate-intensity aerobic exercise has the characteristics of moderate intensity, with the heart rate reaching 70%–85% of the maximum heart rate. It is supplied with energy through a mixture of aerobic and anaerobic metabolism and poses a certain challenge. Resistance exercise, through means such as equipment, elastic bands, or one's own body weight, resists resistance. It can increase muscle mass, enhance strength, and improve metabolism, etc. In addition to these more well-known exercises, some traditional exercises have gradually been widely applied in clinical diagnosis and treatment. Among them, Wuqinxi was created by Hua Tuo, a famous traditional Chinese medicine doctor in China. Its movements are derived from tigers, deer, bears, apes, and birds in nature. Practicing Wuqinxi can mobilize the muscles and bones, dredge the qi and blood, prevent and treat diseases. The strength, speed, and other aspects of its choreographed movements conform to the characteristics of aerobic exercise, but attention also needs to be paid to the regulation of form, spirit, intention, and qi, etc. (79). Tai Chi, as one of the most widely spread traditional exercises, is not a single type of exercise. Instead, it combines the rotation and movement of the human body, following the law of “force originates from the ground and strength is generated from the root”. It can exercise the heart and lungs, move the joints, and is helpful for the control of blood pressure (80, 81).

### 4.2 Prospects

Studies have shown that exercise therapy has a wide range of applicable populations and no toxic or side effects. Notably, it acts on multiple systems simultaneously—including the cardiovascular, metabolic, nervous, and musculoskeletal systems—meaning it not only delays the progression of chronic diseases such as hypertension, diabetes, and arteriosclerosis, but also reduces the risk of cardiovascular events (9, 11, 82).

Building on this understanding of its benefits, the currently known specific mechanisms linking exercise therapy to hypertension prevention and treatment include: promoting the release of nitric oxide (NO), reducing endothelin-1 (ET-1) concentration to improve vascular function; inhibiting sympathetic nerve activity and regulating the renin-angiotensin system; reducing body weight and improving metabolism; and alleviating inflammatory responses (78, 84). However, the specific and complex microscopic mechanisms behind its blood pressure-lowering effect remain unclear. For instance, the differential ways in which different exercise types (such as traditional Chinese exercises and high-intensity interval training) regulate vascular endothelial function and neuroendocrine pathways, as well as the quantitative correlation between exercise intensity, duration, and blood pressure-lowering effect, have yet to form a systematic conclusion.

To address this gap, future research should proceed across three key dimensions. First, in clinical trials, researchers need to further verify the blood pressure-lowering effects of different exercise therapies through randomized controlled trials (RCTs) with larger sample sizes and longer follow-up periods. Crucially, a quantitative model should be established by linking quantifiable indicators—such as cardiopulmonary exercise testing results, inflammatory factors, and vascular endothelial factors—to blood pressure-lowering effects. This model will help analyze the exercise prescription thresholds for patients with different types of hypertension. Second, in basic research, animal and cell experiments can be used to explore the microscopic mechanisms of exercise intervention in lowering blood pressure. Specifically, these experiments can analyze the molecular regulatory networks through which exercise acts on vascular smooth muscle cells and myocardial cells, filling the current gap in microscopic mechanism research. Finally, in future clinical applications, promoting the popularization of heart rate monitoring devices (such as smart bracelets) will enable precise regulation of exercise programs. At the same time, it is necessary to explore the optimal combined programs of exercise therapy with other lifestyle interventions (such as diet adjustment) to further improve patient compliance. Collectively, these future prospects will not only refine the basic theoretical system of exercise therapy but also provide more operable clinical strategies for hypertension prevention and treatment—ultimately reducing the social burden of the disease.

### 4.3 Advantages and limitations of the study

In previous related studies, researchers often focused on comparing exercise modes with different intensities or different training methods, or only focused on traditional Chinese exercises while ignoring the overall concept of exercise therapy. For the first time, this article adopts the overall perspective of exercise therapy to conduct a comprehensive and systematic summary and comparison of various exercise therapies, including aerobic exercise of different intensities, resistance training, yoga, and traditional Chinese exercises. A total of 63 randomized controlled trials (RCTs) were included in the study, and for the first time, studies related to low-intensity aerobic exercise were incorporated, aiming to provide a basis for clinical diagnosis and treatment. On the other hand, the primary outcome indicator of this article is ABPM, which can assess the changing trends and short-term variability of patients' blood pressure under different environments, body positions, and emotional states (83). The ESC Guidelines for the Management of Arterial Hypertension points out that ABPM is capable of diagnosing special hypertension phenotypes such as nocturnal hypertension, transient hypotension, and post-exercise hypertension, as well as identifying white-coat hypertension and masked hypertension (7). Compared with office blood pressure, ABPM has higher specificity and can predict cardiovascular and cerebrovascular events and mortality more accurately (83). Finally, strict screening was carried out for the included literatures in this article, ensuring the quality of the included literatures and enhancing the credibility of the article.

The limitations of this article are as follows. First, publication bias is relatively obvious: all included studies show positive results, but unpublished negative results cannot be ruled out, which may affect the study's reliability. Second, some exercise types have relatively few studies and small sample sizes, requiring further verification. Third, double blinding is difficult to achieve in exercise therapy-related RCTs; inconsistencies in included studies (such as hypertension levels and medication use) have also affected the reliability and accuracy of results to some extent. Fourth, the primary outcome indicator is ABPM. Compared with office blood pressure measurement, ABPM is more costly and may disrupt patients' sleep quality, reducing compliance. Finally, this article did not include studies on combined exercises or direct comparisons of different exercises, which may have impacted the results.

## 5 Conclusion

In conclusion, network analysis results confirm that exercise therapy can significantly reduce 24-hour average blood pressure. Notably, both conventional exercise training (such as aerobic exercise of different intensities, resistance training, etc.) and traditional Chinese exercises (such as Tai Chi, Baduanjin, etc.) exhibit good blood pressure-lowering effects for hypertension patients. Crucially, the conclusions of this study hold practical reference value for clinical application: medical workers can formulate individualized exercise prescriptions based on patients’ specific conditions. For instance, low-intensity aerobic exercise or Tai Chi may be selected for elderly patients or those with poor baseline physical fitness; moderate-intensity aerobic exercise or resistance training can be adopted for young patients or those with good physical fitness; and for patients with other comorbid basic diseases (such as obesity), fat reduction training can be incorporated and combined with lifestyle interventions like dietary adjustment. Through this tailored approach, long-term individualized exercise prescription management for hypertension patients can be effectively ensured. It is worth noting, however, that due to the limitations in the quality and quantity of the included studies, these conclusions still require further verification through high-quality clinical trials.

## Data Availability

The raw data supporting the conclusions of this article will be made available by the authors, without undue reservation.
