# Supplementary material for: Comparing the efficacy of different types of exercise therapy in patients with essential hypertension: a systematic review and network meta-analysis
Source: Front Cardiovasc Med. 2025 Oct 20;12:1604112. doi: 10.3389/fcvm.2025.1604112 (PMC12580293; doi:10.3389/fcvm.2025.1604112)

Supplementary Material S1

Sensitivity Analysis of Systolic Blood Pressure

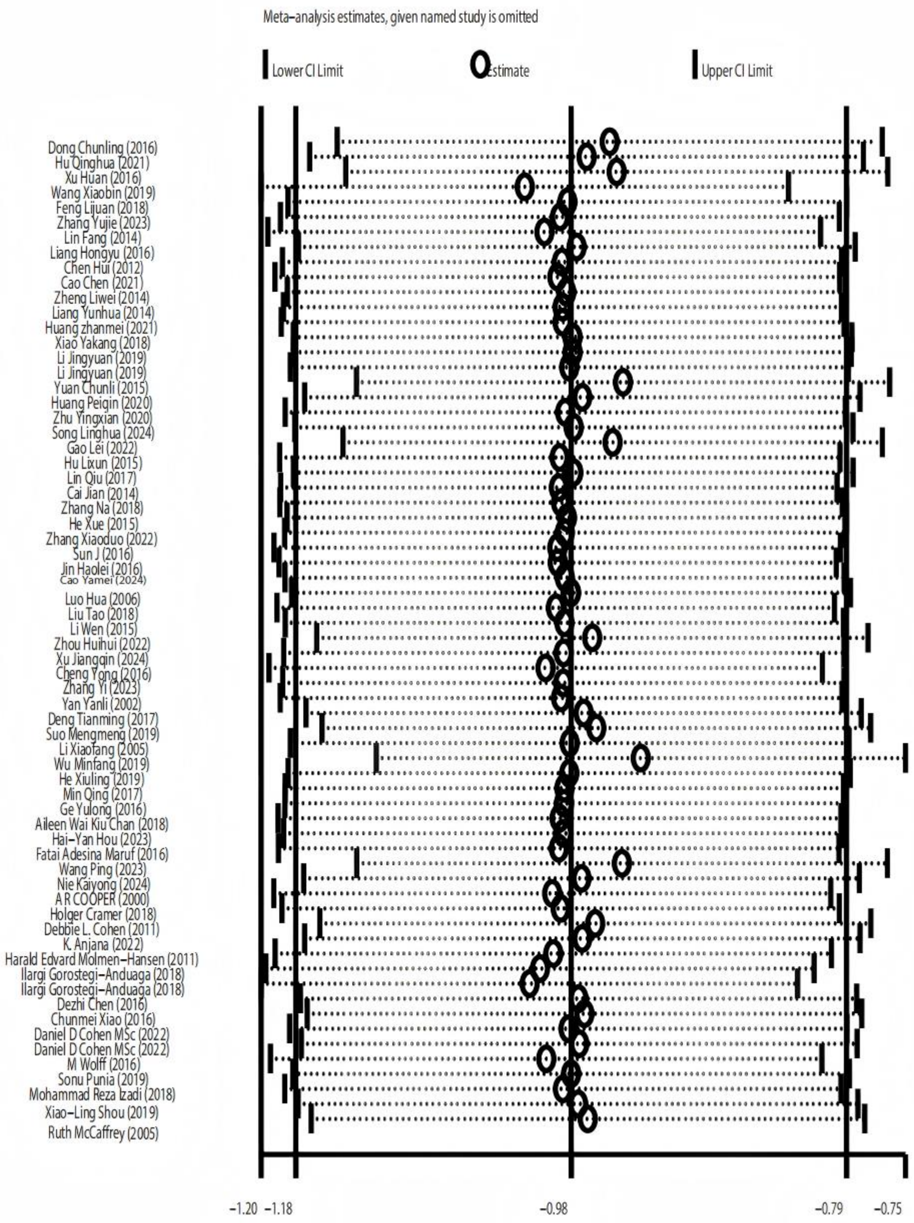

Sensitivity Analysis of Diastolic Blood Pressure

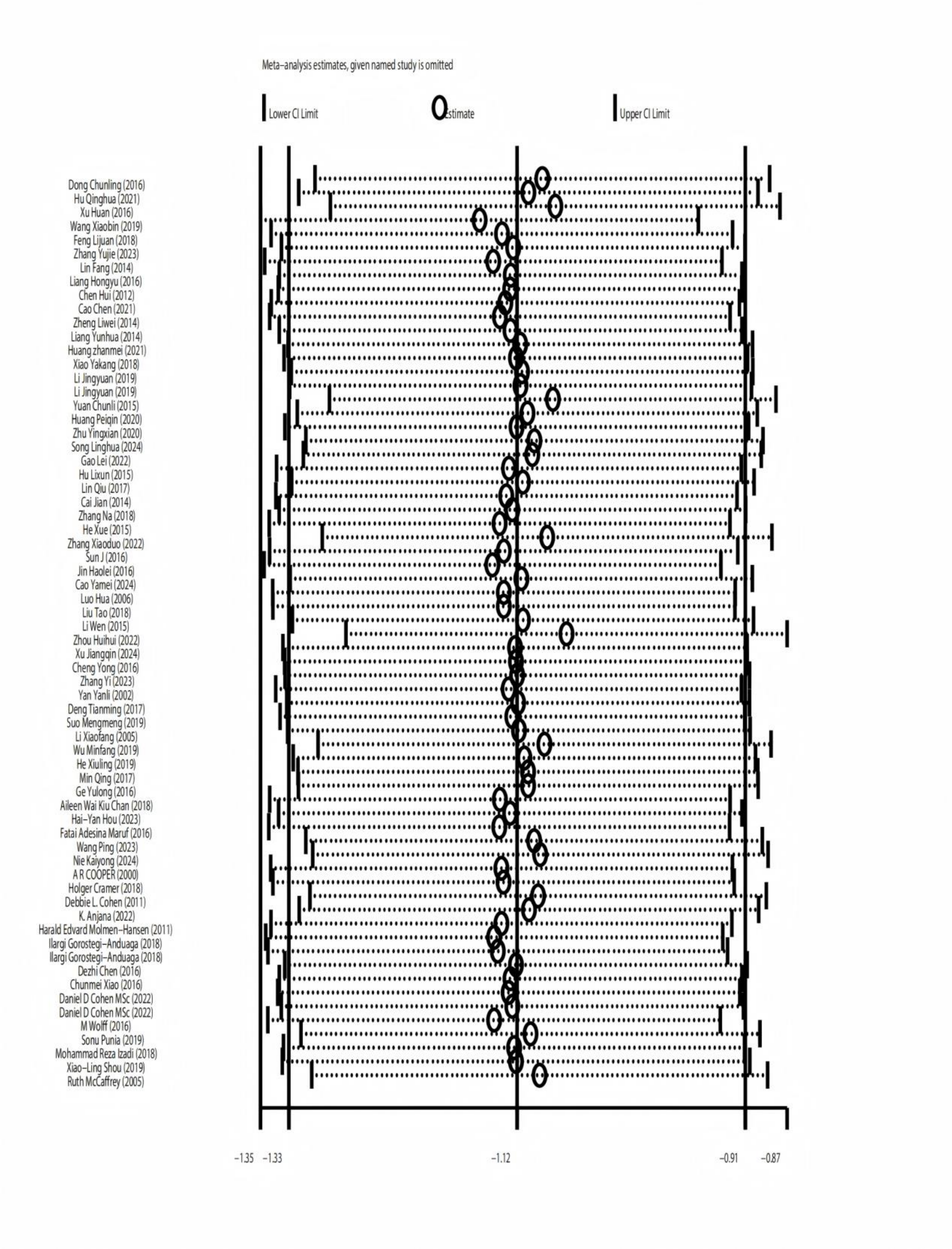

Supplement: Supplementary file 1 [file Datasheet1.pdf]
